# C-met inhibition blocks bone metastasis development induced by renal cancer stem cells

**DOI:** 10.18632/oncotarget.9997

**Published:** 2016-06-14

**Authors:** Lucia D'Amico, Dimas Belisario, Giorgia Migliardi, Cristina Grange, Benedetta Bussolati, Patrizia D'Amelio, Timothy Perera, Ettore Dalmasso, Luca Dalle Carbonare, Laura Godio, Paolo Comoglio, Livio Trusolino, Riccardo Ferracini, Ilaria Roato

**Affiliations:** ^1^ Department of Biomedicine, Cancer Immunology, University of Basel, Basel, Switzerland; ^2^ CeRMS, A.O. Città della Salute e della Scienza, Torino, Italy; ^3^ IRCC, Institute for Cancer Research and Treatment, Candiolo, Torino, Italy; ^4^ Department of Medical Sciences, University of Turin, Torino, Italy; ^5^ Department of Molecular Biotechnology and Health Science, Molecular Biotechnology Center, University of Turin, Torino, Italy; ^6^ Gerontology Section, Department of Medical Sciences, University of Torino, Torino, Italy; ^7^ Janssen Research and Development, Beerse, Belgium; ^8^ Urology Section, A.O. Città della Salute e della Scienza, Torino, Italy; ^9^ Clinic of Internal Medicine, Section D, Policlinico G.B. Rossi Verona, Verona, Italy; ^10^ Department of Pathology, A.O. Città della Salute e della Scienza, Torino, Italy; ^11^ Department of Orthopaedic Oncology, CTO Hospital, Torino, Italy

**Keywords:** bone metastasis, renal cancer, cancer stem cells, c-MET, CCL20

## Abstract

Cancer stem cells (CSCs) are key players in bone metastasis. In some renal tumors CSCs overexpress the HGF receptor c-MET, speculating that c-MET targeting could lead to bone metastasis inhibition. To address this hypothesis we isolated renal CD105+/CD24−CSCs, expressing c-MET receptor from a primary renal carcinoma. Then, to study their ability to metastasize to bone, we injected renal CSCs in NOD/SCID mice implanted with a human bone and we tested the effect of a c-MET inhibitor (JNJ-38877605) on bone metastasis development. JNJ-38877605 inhibited the formation of metastases at bone implant site. We showed that JNJ-38877605 inhibited the activation of osteoclasts induced by RCC stem cells and it stimulated osteoblast activity, finally resulting in a reduction of bone turnover consistent with the inhibition of bone metastases. We measured the circulating levels of osteotropic factors induced by RCC stem cells in the sera of mice treated with c-Met inhibitor, showing that IL-11 and CCL20 were reduced in mice treated with JNJ-38877605, strongly supporting the involvement of c-MET in the regulation of this process. To address the clinical relevance of c-MET upregulation during tumor progression, we analysed c-MET in renal cancer patients detecting an increased expression in the bone metastatic lesions by IHC. Then, we dosed CCL20 serum levels resulting significantly increased in patients with bone metastases compared to non-metastatic ones. Collectively, our data highlight the importance of the c-MET pathway in the pathogenesis of bone metastases induced by RCC stem cells in mice and humans.

## INTRODUCTION

Renal cell carcinoma (RCC) commonly metastasizes to bone, indeed 35% of patients with advanced RCC develop bone metastases [1]. Bone lesions are osteolytic and worsen the prognosis, causing morbidity associated to the skeletal related events, such as pathological fractures, spinal cord compression or hypercalcemia and requirement for surgical treatment or palliative radiotherapy [2–3]. Despite the clinical relevance the mechanism by which RCC preferentially metastasizes to bone is poorly understood. The bone metastatic process requires a series of interactions between tumor cells and bone microenvironment, which release many factors activating bone resorption by osteoclasts (OCs) [4–6]. Recent work essentially focused on production of chemokines and interleukins by the sites of metastasis, which in turn attract cancer cells to explain different tropism for metastatic sites. Classically, this is the case for CCL21/CCR7 involved in lymph node metastasis, CCL27/CCR10 in skin metastasis or CCL20/CCR6 involved in metastases at multiple sites [7–8]. In particular, CCL20 has been produced by different cancer cells as pancreatic, breast and renal carcinoma [9]. Furthermore, high expression of interleukin-11 correlated with poor prognosis in clear-cell renal carcinoma, highlighting the clinical relevance of an early detection of IL-11 and CCL20 in renal carcinoma patients.

Our group recently demonstrated an important role for cancer stem cells (CSCs) in promoting bone metastasis formation in breast and lung cancer, opening the perspective to target CSCs to prevent or block the bone metastatic process [10–11]. Literature reports different attempts to characterize CSCs in RCC, resulting in the identification of CD105, CXCR4 and ALDH1 as the most reliable markers [12–15]. In other tumors as well as RCC, the expression of CD105 is related to the ability of initiating a metastatic process, for instance in breast cancer the subpopulation of cell expressing CD105 correlates with a high migratory ability [16] and in hepatocarcinoma, CD105 promotes the invasion and metastases of liver cancer cells [17].

The characterization of different mutations in some tyrosine kinases (TKs) expressed by RCC lead to the introduction of TK inhibitors in the treatment of RCC [18–19], which replaced immunotherapy as the standard of care for these patients, changing the therapeutic approach of RCC. c-MET, the HGF receptor, is normally involved in cell growth, differentiation and neo-vascularization, but its dysregulation has been implicated in tumor formation, invasion and angiogenesis [20]. In hereditary and sporadic papillary RCC patients activating mutations of c-MET have been identified [21–23] and over expression of c-MET has been also reported in clear cell carcinoma [24–26].

Since c-MET mediates the interaction between cancer cells and mesenchymal cells of the bone microenvironment [27], we propose it as a suitable candidate to disrupt bone metastatic process. TK and more specific MET inhibitors have been tested and clinical trials are ongoing for the treatment of RCC [28–29]. JNJ-38877605 is a highly selective c-MET ATP competitive kinase inhibitor [30], which induces cell death in tumor cells overexpressing c-MET protein or expressing constitutively activated c-MET protein. The expression of c-MET have been described on a RCC cell line derived from human bone [31], but its expression on RCC stem cells have not been investigated. Here we studied the ability of RCC stem cells to metastasize bone through a human-in-mice model of bone metastases, where a small fragment of human bone was implanted sub-cutis in NOD-SCID mice [10–11, 32–33], thereby providing a viable and active human microenvironment. Moreover, we reported the relevant role of c-MET in the bone metastatic process induced by RCC stem cells. Indeed, we were able to block the bone metastasis formation in mice by inhibiting c-MET with a selective and specific inhibitor, JNJ-38877605. Finally, c-Met expression was increased in renal cancer patients with bone metastasis and CCL20 was upregulated in the circulation of the same patients supporting the relevance to target c-Met to reduce renal cancer-derived bone metastases.

## RESULTS

### RCC stem cells express high level of c-MET

**Figure 1:**
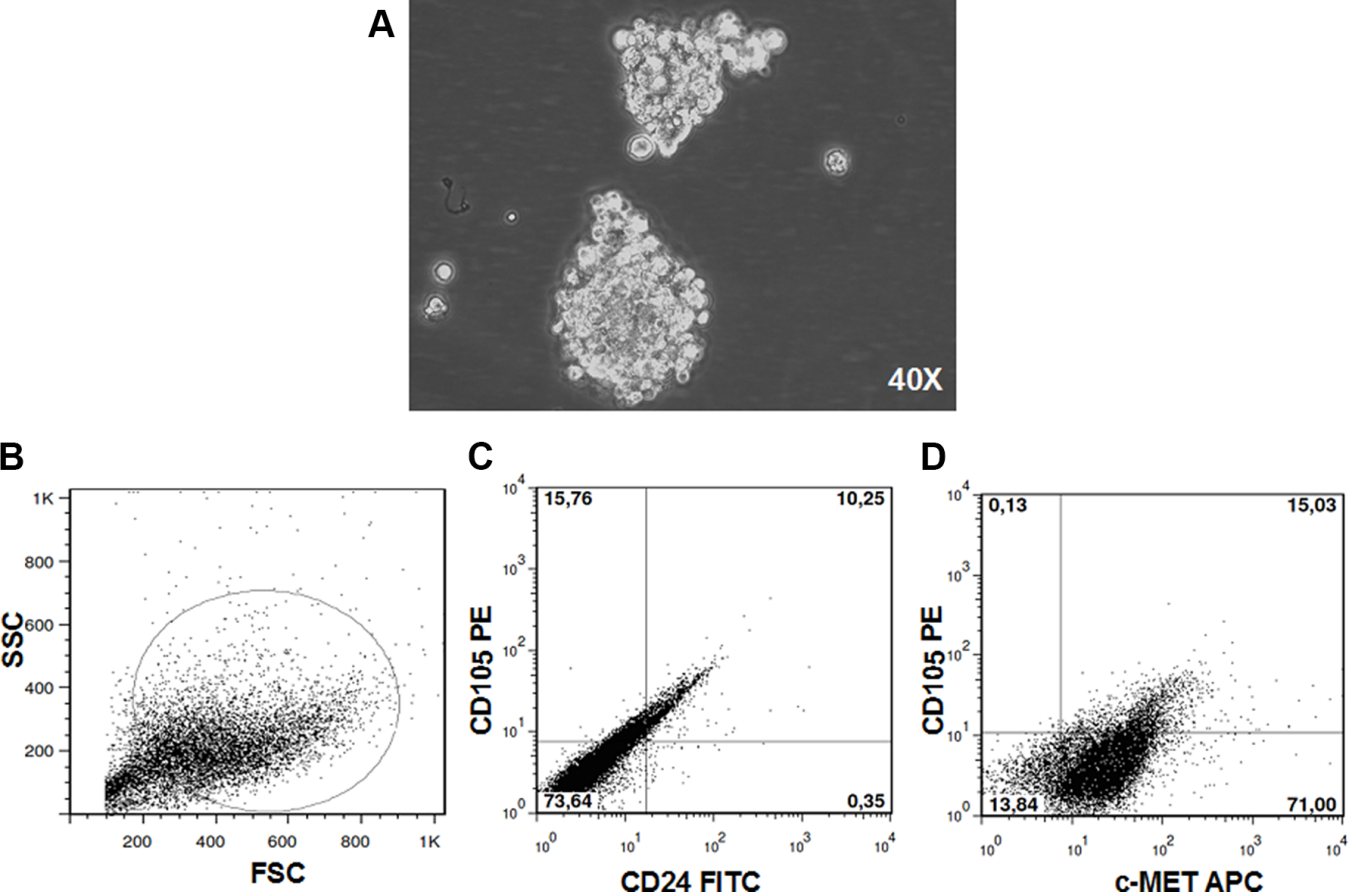
RCC stem cells CD105+CD24− RCC stem cells previously isolated from a primary clear cell renal carcinoma grew as spheroid (magnification × 40) (**A**). After SC injection these cells originated a tumor mass that recapitulated the heterogeneity of the primary tumor (**B**), including a subpopulation of RCC stem cells (**C**) expressing c-MET (**D**).

RCC stem cells were previously isolated from a primary clear cell renal carcinoma as CD105+CD24−cells and tested for stemness features. The negative counterpart CD105-CD24-cells did not show tumorigenic activity after injection in SCID mice [12]. We transduced CD105+CD24− RCC stem cells with a luciferase-expressing lentiviral vector and grew them as spheroids (Figure 1A). After SC injection these cells originated a tumor mass that recapitulated the heterogeneity of the primary tumor (Figure 1B), including a subpopulation of RCC stem cells expressing CD105 and c-MET (Figure 1C–1D). The percentage of CD105+/CD24−/c-MET+ cells was 65%.

### RCC stem cells metastasize to human bone

**Figure 2:**
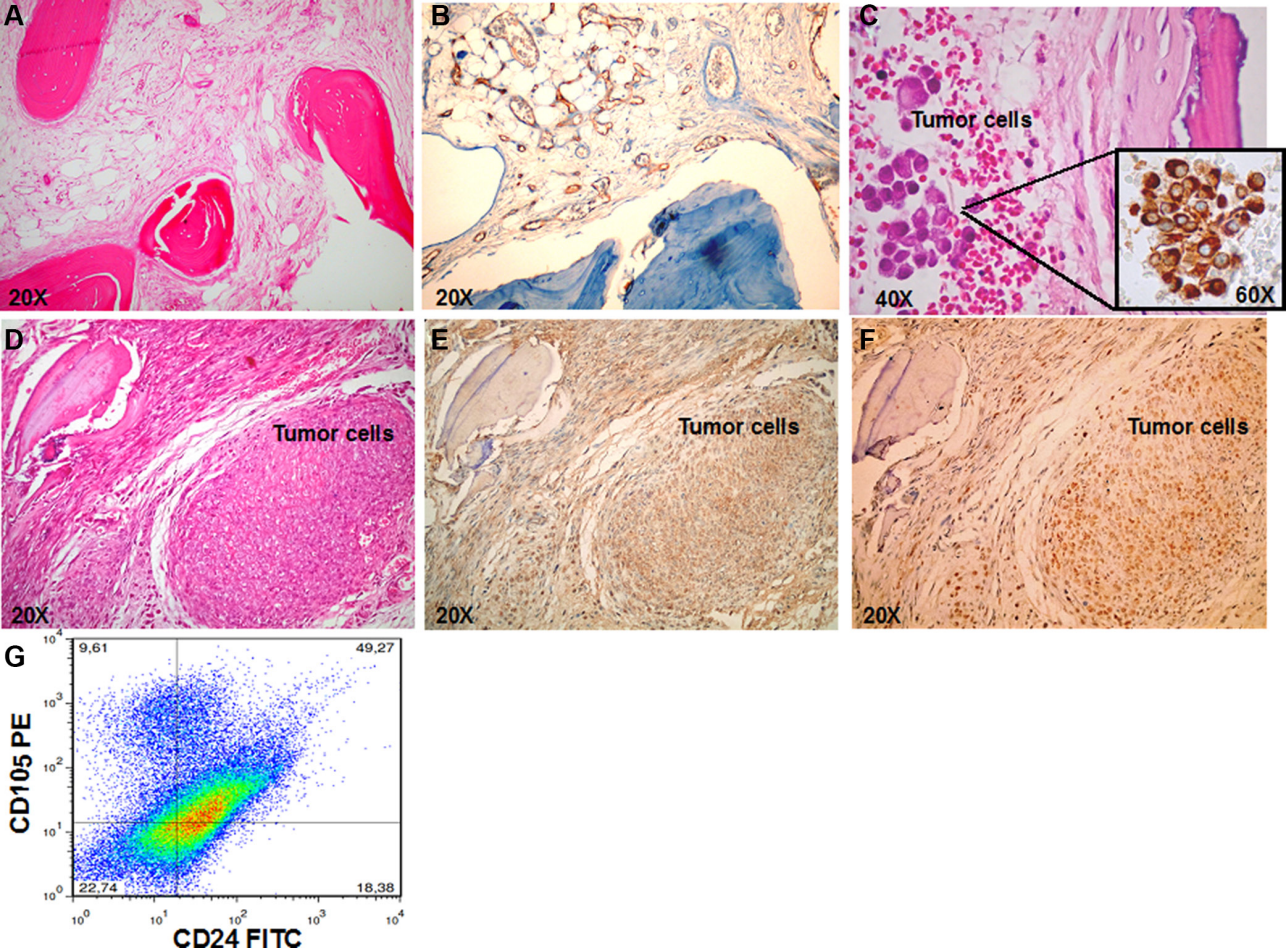
RCC stem cells efficiently metastasize to bone (**A**) H&E-stained section in control mice show the viable human bone (magnification × 20). (**B**) IHC staining for human CD34 confirmed the presence of human vascularization (magnification × 20). (**C**) H&E and IHC staining for vimentin confirmed the presence of bone metastasis in mice injected with RCC stem cells through IC route (magnification × 20 and × 60). (**D**–**F**) Serial slices of the human implanted bone show bone metastasis in mice injected SC with RCC stem cells. In the tumor lesions cells express both CD105 (E) and c-MET (F) (magnification × 20). (**G**) Flow citometry analysis of cells retrieved by bone shows a cell population resembling the heterogeneity of the primary tumor, with about 10% of CD105+CD24− RCC stem cells.

To determine the ability of RCC stem cells to directly induce bone metastases, we injected them subcutaneously (SC), close to the bone implant or through intracardiac route (IC) in NOD/SCID mice carrying a small piece of human bone, previously implanted in a flank. The number of mice in the experimental groups, time of mice survival, percentages of bone engraftment, and numbers of lung and bone metastases are indicated in Table 1. The human bone implants resulted viable, with mineralized areas, stromal cells and little or no necrosis evident in the grafts (Figure 2A). Human neo-vascularization was present, with numerous vessels expressing human CD34 (Figure 2B). RCC stem cells colonized human implanted bone after either IC or SC injection (Figure 2C–2D). IHC analysis of serial slices showed that tumor cells in bone lesions (Figure 2D) expressed CD105 and c-MET (Figure 2E, 2F). By isolating tumor cells from the implanted bone, we retrieved a cell population resembling the heterogeneity of the primary tumor, with about 10% of CD105+CD24− RCC stem cells (Figure 2G). To rule out metastatic seeding of RCC stem cells in bones of the hosts, we performed X-rays on the mice without detecting lesions in mouse bones, demonstrating a species-specific tropism of these cells to human bone (Figure S1).

**Table 1:** *In vivo* results following JNJ treatment in tumor bearing mice

Mice group	*N*	Bone engrafment	Death	Lung metastasis	Bone metastasis
**Bone+CSC+veh**	31	30/31 (96,8)	3/31 (9,6%)	7/28 (25%)	15/28 (53,6%)
**Bone+CSC+JNJ**	19	19/19 (100%)	2/19 (10,5%)	4/17 (23,5%)	0/17
**Bone+veh**	11	10/11 (90,1)	1/11 (9%)	0/10	0/10
**Bone+JNJ**	11	10/11 (90,1)	2/11 (18,2)	0/9	0/9
**CSC+veh**	6	no bone implant	1/6	1/5	
**CSC+JNJ**	6	no bone implant	0/6	1/6	

### RCC stem cells develop bone metastases with a c-MET dependent mechanism

**Figure 3:**
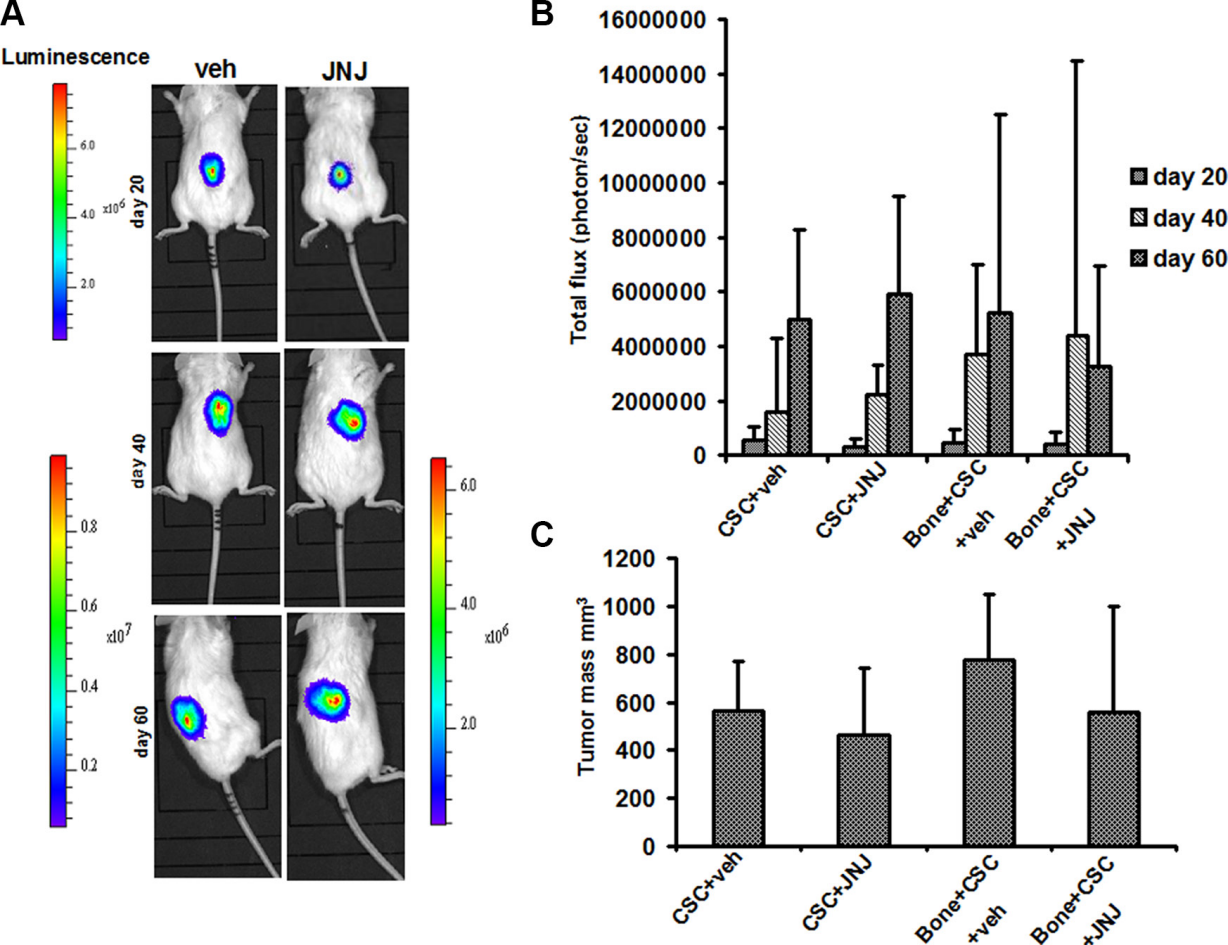
RCC stem cell growth and *in vivo* localization (**A**) Tumor growth was examined by BLI. Representative images of two animals after SC injection of RCC stem cells, at different time points (20, 40, 60 days). (**B**) At 20 days the primary tumors were macroscopically evident and the luciferase signal increased progressively over the course of the study. (**C**) JNJ did not significantly affect tumor growth and the endpoint tumor mass volume.

**Figure 4:**
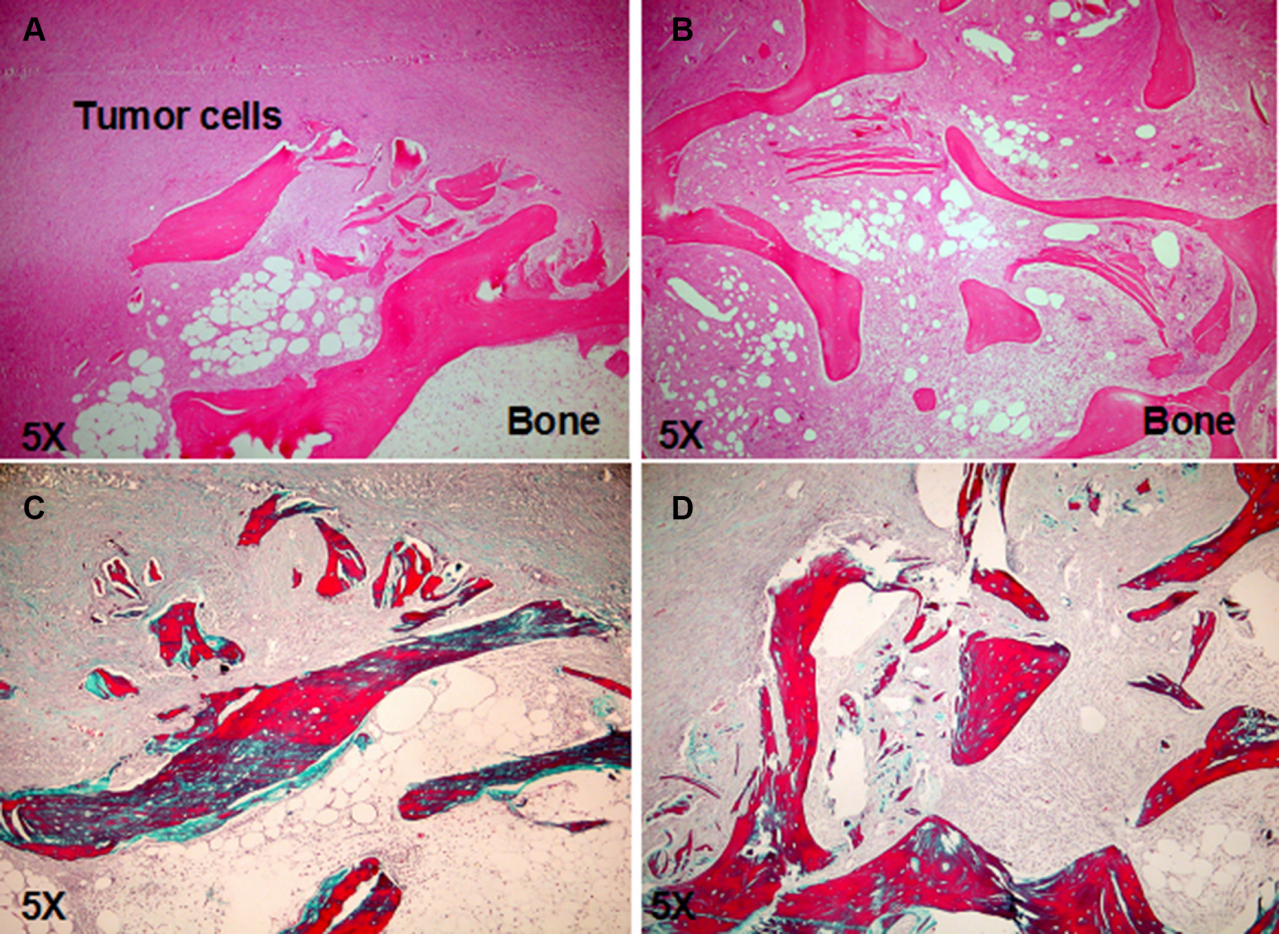
JNJ inhibits bone metastasis formation (**A**) H&E stained section showed bone lesion in untreated mice, (**B**) whereas no bone metastasis was present in JNJ-treated mice. (**C**) The trichrome staining showed an increased bone remodeling due to bone metastases, with a larger area of new bone apposition consequent to the increased metastatic bone resorption in untreated mice (blue stain) compared to JNJ-treated ones (**D**) (magnification × 5).

To investigate whether c-MET has a role in the bone metastatic process induced by RCC stem cells, we treated mice with JNJ-38877605 (hereafter referred to as JNJ), a highly specific c-MET inhibitor, which was not toxic on RCC stem cell culture *in vitro*, also at high concentrations as demonstrated by the citoxicity assay (Figure S2). We monitored the growth and localization of these cells through IVIS, at different time points for 60 days. At 20 days the primary tumors were macroscopically evident and the luciferase signal increased progressively over the course of the study. Importantly, JNJ did not grossly affect tumor growth, with only a slight reduction in the first 20 days after implantation followed prompt alignment with untreated controls (Figure 3A). This result was also confirmed by the quantification of the mean luciferase intensity (Figure 3B) and of the endpoint tumor mass volume (Figure 3C). Of note, while JNJ treatment did not influence overall tumor growth kinetics, it manifestly inhibited the formation of metastases at bone implant site. Indeed, bone lesions were present in untreated mice, but they were absent in JNJ-treated mice (Figure 4A, 4B). The marked increase in bone remodeling due to bone metastases was evaluated through trichrome staining, which showed a larger area of new bone apposition consequent to the increased metastatic bone resorption in untreated mice (blue stain, Figure 4C) compared to JNJ-treated ones (Figure 4D).

Altogether these results support our hypothesis that RCC stem cells are osteotropic and induce bone metastases through the c-MET pathway involvement.

### JNJ blocks bone metastasis by inhibiting stem cell-induced OC activation

**Figure 5:**
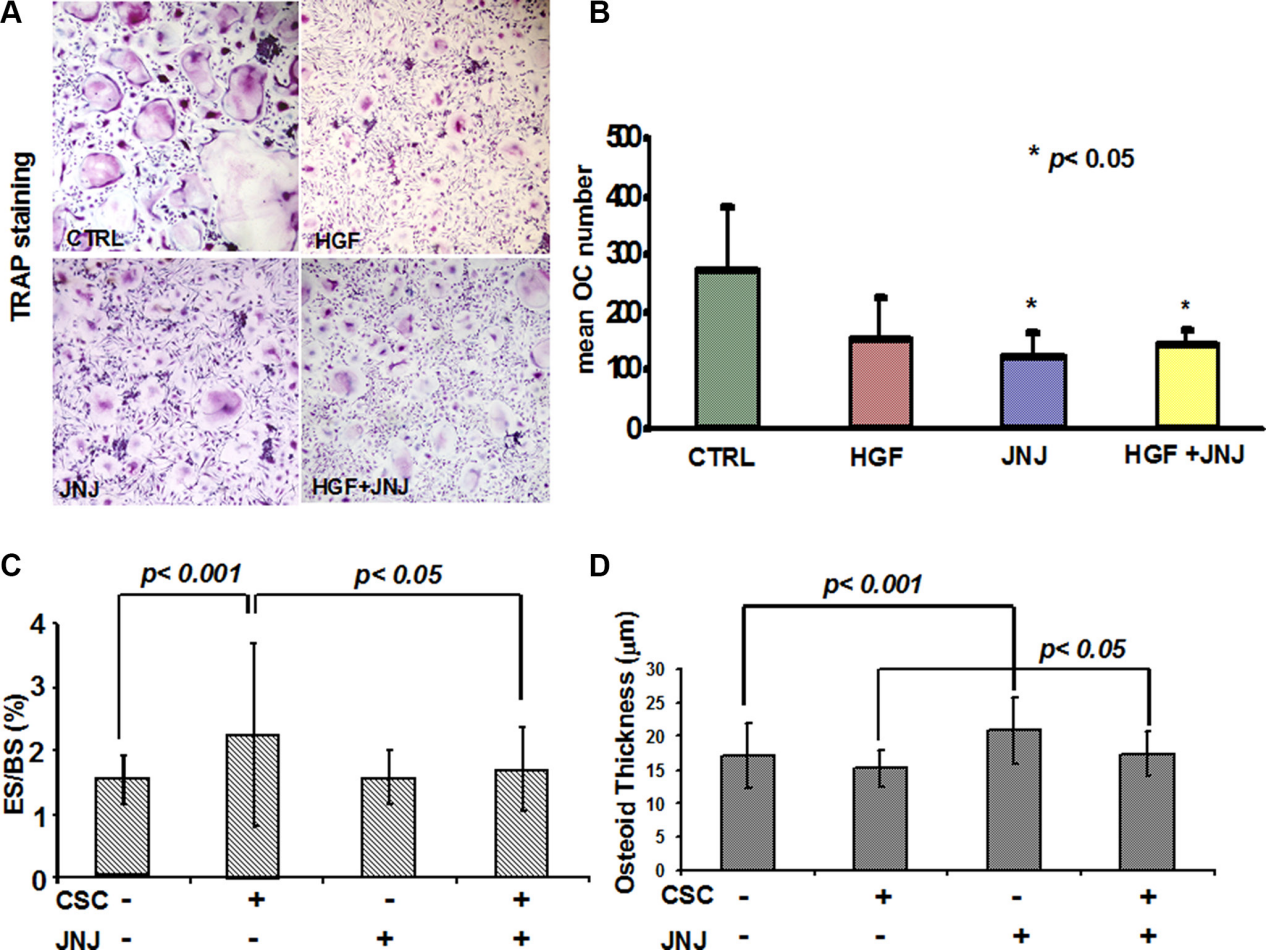
Effects of JNJ on osteoclasts (OCs) (**A**) Human PBMCs cultured under pro-osteoclastogenic conditions differentiated into large, multinucleated and TRAP+ OCs, whereas JNJ treatment inhibited OC differentiation. (**B**) The number of OCs was significantly reduced by the JNJ treatment. (**C**) Histomorphometric analysis on human bone implants showed that RCC stem cells caused a significant increase of OC activity (percentage of erosion surface) in mice injected with RCC stem cells compared to control mice (animals not injected with RCC stem cells), suggesting a direct stimulation by RCC stem cells on the OC bone resorption activity. JNJ significantly decreased OC activity induced by RCC stem cells, *p* < 0.05. (**D**) RCC stem cells did not cause significant changes in osteoblast activity (osteoid thickness), while JNJ increased it, showing an anabolic effect *p* < 0.05. ES: erosion surface, BS: bone surface.

Since renal cancer bone metastases are characterised by an increased bone resorption activity, which is mainly dependent on OCs, and considering that the c-MET/HGF pathway is involved in osteoclastogenesis, we investigated the effect of JNJ on OCs *in vitro*, thus avoiding interferences of bone microenvironment and RCC stem cells. In cultures of human PBMCs kept under pro-osteoclastogenic conditions, we added JNJ showing that it significantly reduced both the formation of large, multinucleated and TRAP+ OCs (Figure 5A) and the number of OCs compared to the control (Figure 5B).

Beside the effect played on OCs *in vitro*, we addressed the effect of JNJ *in vivo* by performing histomorphometric analysis on human bone implants and on mice bones. In untreated mice, RCC stem cells caused a significant increase of OC activity as demonstrated by the augmented percentage of erosion surface, *p* < 0.001 (Figure 5C), suggesting a direct stimulation by RCC stem cells on the OC bone resorption activity. The treatment with JNJ resulted in a significantly decreased OC activity in mice injected with RCC stem cells, *p* < 0.05 (Figure 5C), whereas JNJ did not interfere with OC activity in control mice. This result suggests that JNJ indirectly affects OC bone resorption activity, because it interferes with RCC stem cell molecular signals, which stimulate OC activity. When coupled with the *in vitro* data, these results suggest that Met inhibition has a direct negative effect on OC differentiation and an indirect effect on OC activity, likely mediated by perturbation of paracrine signals from tumor cells.

Since c-MET/HGF pathway is also known to regulate osteoblasts (OBs), we investigated the effect of JNJ on OB activity *in vivo*. We observed that RCC stem cells did not cause significant changes in the osteoid thickness, while JNJ increased it, *p* < 0.05 (Figure 5D). These results suggest that JNJ treatment has an anabolic effect, strengthening the relevance to block the c-MET pathway in order to contrast the bone metastatic commitment of RCC stem cells.

### JNJ treatment reduces the level of circulating osteolytic factors

To investigate the role of c-MET in promoting the bone metastatic process we measured the circulating levels of osteotropic factors induced by RCC stem cells in the sera of mice treated with c-Met inhibitor JNJ. Interestingly we were able to quantify the expression in tumor bearing mice of the human chemokines IL-11 and CCL20, known as key players in the regulation of cancer cell migration and progression from different solid tumors [34–36].

**Figure 6:**
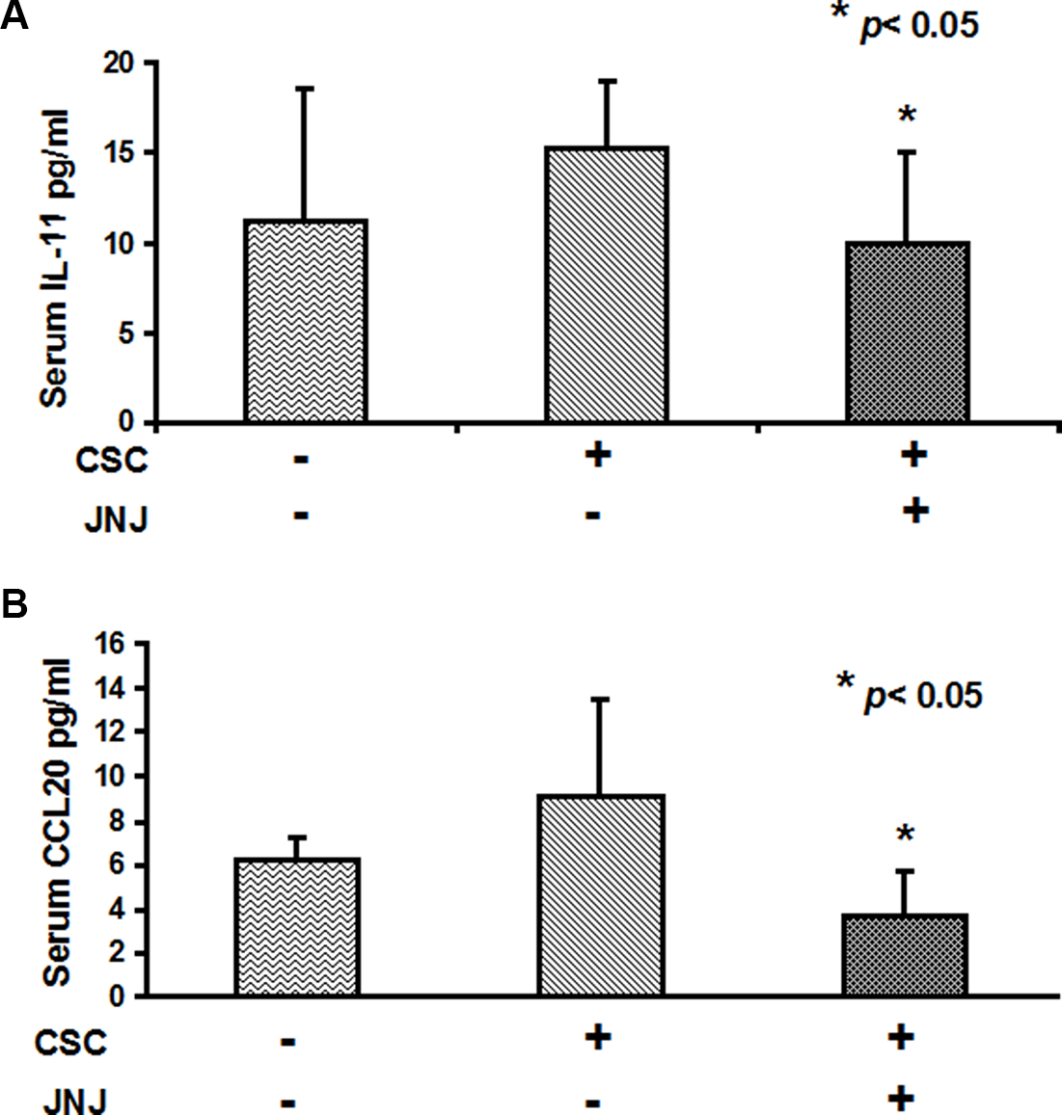
IL-11 and CCL20 serum levels are reduced by JNJ treatment (**A**) Human IL-11 and (**B**) CCL20 serum levels resulted significantly higher in mice untreated with JNJ than in treated ones, *p* < 0.05.

Importantly, JNJ treatment significantly reduced human IL-11. CCL20 levels resulted significantly higher in mice untreated with JNJ than in treated ones, *p* < 0.05 (Figure 6A–6B), strongly supporting the involvement of cMet in the regulation of the bone metastatic process.

### c-MET and CCL20 expression are increased in renal cancer patients with bone metastases

**Figure 7:**
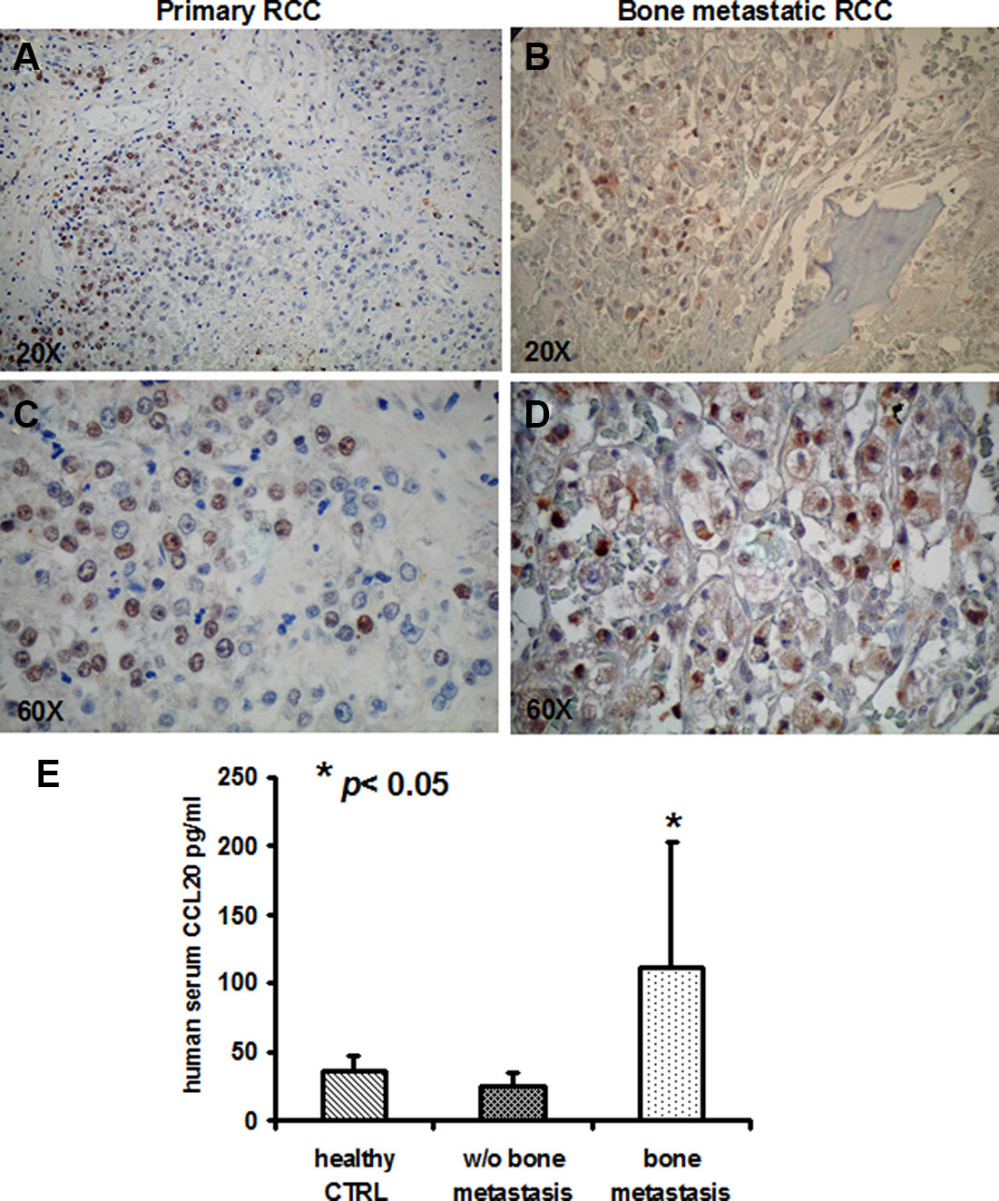
c-MET and CCL20 expression in human samples (**A**, **C**) c-MET expression in primary RCC and (**B**, **D**) in bone metastastic samples (magnification × 20 and × 60). (**E**) CCL20 serum levels resulted significantly increased in the sera of patients with bone metastases compared to non-metastatic ones, *p* < 0.05.

To further confirm our previous data on humans, we analysed c-Met expression in IHC of tumor samples from patients with primary renal carcinoma and bone metastases, the clinical data of patients are reported in Table S1. c-Met was detected on 9/12 primary tumors and in all (4/4) bone metastatic samples as shown in (Figure 7A–7D). In parallel, to further address the clinical relevance of c-Met upregulation during tumor progression, we dosed CCL20 in the serum of renal cancer patients with and without bone metastases and in healthy controls. CCL20 levels significantly increased in the sera of patients with bone metastases compared to non-metastatic ones, *p* < 0.05 (Figure 7E). All together these data sustain the clinical relevance of targeting c-Met as possible marker of renal cancer dissemination to the bone.

## DISCUSSION

The cellular mechanisms underlying tumor heterogeneity are subject of intense research in the cancer biology field. Indeed, cells within the tumour population itself often exhibit functional variety with distinct proliferative, differentiative and metastatic capacities.

Over recent years increasing data indicated the existence of CSCs in multiple solid tumors and compelling evidences highlight their role in promoting tumor dissemination to distant organs and in particular to bone [10–11]. A number of cell surface markers as CD133, CD44 or CD24 have been identified and utilized for the isolation of subsets enriched for CSCs [37–38]. In renal cancer, stem cells have been characterized as CD105+CD24− cells [12] and here we demonstrate that RCC stem cells directly metastasize to bone in NOD/SCID mice, previously implanted with a small fragment of human bone [33]. Bone metastasis derives by a complex cross-talk between cancer cells and bone microenvironment, thereby the presence of a “human pre-metastatic niche” as an implanted human bone is important to address the mechanisms leading tumor progression to bone [39–40].

Recent findings indicate that the MET tyrosine-kinase receptor is a sensor of adverse microenvironmental conditions (such as hypoxia) and drives cell invasion and metastasis [41]. In particular, it is involved in the bone metastatic process, because it is a mediator of the interactions between tumor cells and the bone microenvironment in breast cancer [27]. c-MET is widely expressed in human cancers [42] and also in CSCs isolated from different tumors such as glioblastoma [37], head and neck carcinoma [43], pancreatic [44] and colorectal cancer [45–46]. Here we report c-MET expression on CD105+ CD24− RCC stem cells which, implanted SC in NOD-SCID mice, recreate the heterogeneity of the primary tumor, including a subpopulation of RCC stem cells expressing CD105 and c-MET. Our data suggest that c-Met expression on RCC stem cells drives renal cancer progression to bone, since c-Met+ RCC stem cells directly formed bone lesions. Furthermore, a selective c-MET inhibitor treatment abrogated bone metastases development, indicating that HGF/c-MET signalling is relevant in the metastatic process induced by RCC stem cells. c-MET has been found to be highly expressed in prostate cancer bone lesions [47], and a c-MET inhibitor reduced bone metastasis progression in breast cancer [48]. Thus, our data confirm now a role for c-MET in the RCC-induced bone metastatic process, suggesting the relevance of targeting CSCs in order to avoid disease relapse and particularly development of bone metastases.

A characteristic of bone metastases from renal cancer is the presence of pure lytic lesions due to OC activation, accompanied by a striking neo-vascularization and by an entire substitution of the bone tissue with tumor cells. Here, we observed that RCC stem cells grow in the human implanted bone, where they prompt OC activation without affecting OB activity. JNJ inhibited the stimulatory activity of RCC stem cells on OCs, because it likely interferes with paracrine factors produced by RCC stem cells, which promote OC bone resorption. JNJ also affects OC differentiation and stimulates OB activity, thus it has an anabolic effect. This latter point is particular relevant because JNJ inhibits the bone metastasis formation and concomitantly it has a protective effect on bone through an anabolic action. As confirmation of a direct effect of c-MET inhibitors on bone metastases and bone cells, literature data reported clinical and pre-clinical evidences that cabozantinib (a tyrosine kinase inhibitor targeting c-MET and VEGFR-2) has a double antitumoral and anti-bone resorptive effects in patients with advanced RCC [49]. Moreover, Santini et al. demonstrated that cabozantinib directly inhibits osteoclasts and reduces RANKL expression on osteoblasts [50].

Other than the effect of JNJ on RCC stem cells and on bone cells, we reported that this c-MET inhibitor interferes with serum levels of IL-11 and CCL20. IL-11 is a negative prognostic factor in renal cancer [34]; consistently, we observed that IL-11 levels were higher in sera of mice with bone metastasis than in non-bone metastasis ones. CCL-20 is expressed by different tumors [35–36, 44], where it contributes to cancer cell growth and *in vivo* invasion [45–46]. The link between CCL20 and CSCs-induced bone metastasis is not known. Here we showed that JNJ treatment reduced CCL20 levels in the sera of treated animals, which did not develop bone metastases, compared to the untreated mice. Finally, for a clinical perspective, we reported that CCL20 levels were higher in sera of renal cancer patients with bone metastases than in non-bone metastatic patients, suggesting a potential role of CCL20 in the bone metastases induced by RCC stem cells. Moreover, T regulatory (Treg) cells, infiltrating renal cancer, express high level of CCR6 [51] and its ligand CCL20 is expressed on tumor cells [48], representing a homing mechanism for Treg that favours the tumor immune escape. Although the function of CCL20 in RCC stem cell-mediated bone metastasis needs further investigation, it is tempting to speculate that blocking CCL20 signaling could be useful to inhibit both bone metastases and tumor immune escape.

In conclusion, we provide evidence for the ability of RCC stem cells to metastasize bone and we report the relevant role of c-MET in the bone metastatic process induced by RCC stem cells in mice and humans. Indeed, we were able to block the bone metastasis formation in mice by inhibiting c-MET with a selective and specific inhibitor.

Out of these findings strongly emerges the relevance to target CSCs in order to avoid tumor metastases. Thus, an integrated approach with the development of suitable *ex vivo* and *in vivo* models should lead to further characterization of the CSCs. High-resolution imaging technology together with the identification of stromal markers will improve our understanding of CSCs to more accurately recapitulate the niche of tumorigenic cells and to address novel mechanisms that operate in CSCs during tumor progression.

## MATERIALS AND METHODS

### The human-in-mice model of bone metastasis

Experimental animals were treated according to the national and international guidelines (the Italian legislative decree 116/92 and the European Community Directive 86/609 CEE) and with the authorization provided by the Italian Ministry of Health (as of D.M. 44/1994-A and subsequent integrations). All of the orthotopic xenograft models were established in NOD/SCID mice as previously described [33]. A small fragment of human bone, derived from the discarded femoral head of an adult patient submitted to total joint replacement (after the patient's informed consent) was transplanted subcutaneously in the left flank of 72 NOD/SCID 4-week-old female mice (Charles River Laboratories Italia). The animals were divided in 3 groups: i) mice with bone implant, injected with RCC stem cells and treated with JNJ (19) or untreated (31); ii) mice with only bone implant treated with JNJ (11) and untreated (11); iii) mice only injected with RCC stem cells treated with JNJ (6) and untreated (6). RCC stem cells were injected subcutaneously (SC) close to the bone implant in 25 mice and by intracardiac (IC) route in 12 mice. For SC injections, 2,0 × 10^4^ RCC stem cells were resuspended in PBS and Matrigel 1:2 (BD Biosciences) and injected in a volume of 100 μL using a 25-gauge needle. For the IC route, 1 × 10^3^ RCC stem cells were injected in the left ventricle. The day after the RCC stem cell injection, we started a systemic treatment with JNJ by gauvage; the compound was administered at a daily dose of 40 mg/kg according to previous tested dose [52]. For 60 days, the development of tumor masses and metastases to bone and other organs was monitored by *In Vivo* Imaging System (IVIS). One of the parameter evaluated to determine the viability of the implant bone was the presence of circulating human IgG in mice sera by mean of a human IgG ELISA, purchased by ICL Inc.

### Lentivirus production and RCC stem cell transduction

The production of lentivirus vector and RCC stem cell transduction were previously described [10]. Briefly, vector stocks were produced by transient transfection of the luciferase transfer plasmid, the packaging plasmids pMDLg/pRRE and pRSV.REV, and the vesicular stomatitis virus (VSV) envelope plasmid pMD2.VSV-G in 293T. The viral supernatants were filtered and viral particles were concentrated by ultracentrifugation according to Follenzi et al. [53]. The viral p24 antigen concentration was determined by HIV-1 p24 Core profile ELISA (Perkin-Elmer Life). The high-titer lentiviruses were added to RCC stem cells, which were incubated for 4 hours. RCC stem cells were tested for luciferase expression through the Luciferase Assay System (Promega Corp.), as described in the protocol kit. Importantly, we did not observe significant differences in tumor phenotypes associated with lentiviral transduction, as demonstrated by histological appearance and flow cytometry analysis (data not shown).

### *In vivo* bioluminescence imaging

To perform IVIS analyses, mice were anesthetized by isoflurane inhalation then intraperitoneally injected with 15 mg/mL D-luciferin (Caliper Life Science). At various time points after tumor implantation (20, 45 and 60 days), the bioluminescence signals were monitored using the IVIS system 2000 series (Xenogen Corp.) consisting of a highly sensitive cooled CCD camera. Two kinetic bioluminescent acquisitions were collected between 0 and 20 min after D-luciferin injection to confirm the peak photon emission, which was recorded as maximum photon efflux per second; imaging times ranged from 1 to 60 sec, depending on the amount of luciferase activity. Data were analyzed using the total photon flux emission (photons/second) in the regions of interest (ROI) defined manually.

### Isolation of bone metastatic cells

After 60 days, the mice were sacrificed, the human implanted bones were retrieved, finely minced and then digested by incubation for 30 min at 37°C in D-MEM containing collagenase I (Sigma-Aldrich) to isolate metastatic cells from the osteolytic lesions. After collagenase neutralization, the cells were washed by Hank's balanced saline solution (Lonza), and red blood cells were lysed with Red Blood cell Lysis Solution (Promega). The cell suspension was forced through a graded series of meshes to separate the cell components from the stroma and aggregates. After filtration, to analyze the phenotype of these bone-derived cells, they were counted and stained for flow cytometry analysis with anti-human CD105PE (Invitrogen) and CD24FITC (BD Pharmingen). For every antibody, we also used the relative isotype control. Samples were analyzed in a FACs Calibur instrument and elaborated by Flowjo (Treestar).

Single cells were also plated in a serum-free DMEM-F12 selective medium (Gibco, Invitrogen) supplemented with 10 ng/ml basic fibroblast growth factor (b-FGF), 20 ng/ml epidermal growth factor (EGF) (PeproTech), 5 μg/ml insulin and 0,4% bovine serum albumin (Sigma-Aldrich). After one week, sphere formation was observed. Renal spheres were collected by gentle centrifugation, disaggregated with a Non-enzymatic Cell Dissociation Solution (Sigma-Aldrich), then stained with CD105 and CD24 to control the phenotype by FACs analysis.

### Immunohistochemistry and histological analysis

Immunohistochemistry was performed on tissues fixed in 10% neutral buffered formalin, and bone tissues were decalcified with EDTA treatment until soft. Tissues were embedded in paraffin, and sections were deparaffinized, rehydrated through graded alcohols and subjected to antigen retrieval for immunohistochemistry. Sections were stained for H&E for morphological studies. The presence of human vessels was demonstrated by staining for anti-CD34 (clone QbndN/10) from Neomarkers, whereas tumors cells were stained by vimentin (clone R9) from DAKO, CD105 (clone 3A9) from Novus Biologicals and c-MET (clone C28) from Santa Cruz Biotechnology, Inc. To identify the collagen fibers on the new bone, a trichrome stain was performed by Gomori's trichrome stain kit (DAKO). TRAP staining was performed to identify osteoclasts, according to the manufacturer's instructions (Roche).

### Histomorphometry

The samples were fixed in 70% ethanol and embedded undecalcified in methyl-methacrylate resin (Merck 800590, Germany). Bone sections were cut by using a microtome (Polycut S, Leica Microsystems) equipped with a carbide-tungsten blade, stained with Goldner's stain, and mounted on microscope slides for histomorphometric measurements. The sections were obtained from three different levels of the methyl-methacrylate block, each separated by a thickness of 250 μm. Histomorphometric results were calculated as the mean of the values obtained from the three different levels as an approximation of a 3-D evaluation. This also avoids replicating the sampling of any single bone remodeling unit.

Measurements were performed by means of an image analysis system consisting of an epifluorescent microscope (Leica DM2500) connected to a digital camera (Leica DFC420 C) and a computer equipped with a specific software for histomorphometric analyses (Bone 3.5, Explora Nova).

Histomorphometric parameters were reported in accordance with the ASBMR Committee nomenclature [54]. All thickness/depth results (O.Th, MAR, E.De, W.Th) were corrected for obliquity of sections by multiplying by π/4.

### Serum detection of IL-11 and CCL20

To analyze the expression of human IL-11 and CCL-20 in mice, we utilized the multi-analyte detection system Milliplex Map, according to the manufacturer's instructions. Molecule detection was performed with the instrument Luminex^®^ 200^™^ (Luminex Corporation), and data were analyzed through the MILLIPLEX analyst software.

### Statistical analyses

Statistical analyses on multiple simultaneous comparison were performed by one way ANOVA, whereas differences between two groups were performed by Student's *t*-test with the Statistical Package for the Social Sciences (spssx/pc) software (SPSS, Chicago, IL, USA). The results were considered statistically significant for *p* < 0.05.

## SUPPLEMENTARY MATERIALS FIGURES AND TABLE


