# Supplementary material for: C-met inhibition blocks bone metastasis development induced by renal cancer stem cells
Source: Oncotarget. 2016 Jun 14;7(29):45525–37. doi: 10.18632/oncotarget.9997 (PMC5216739; doi:10.18632/oncotarget.9997)
Supplement: Supplementary file 1 [file oncotarget-07-45525-s001.pdf]

C-met inhibition blocks bone metastasis development induced by renal cancer stem cells

Supplementary Materials

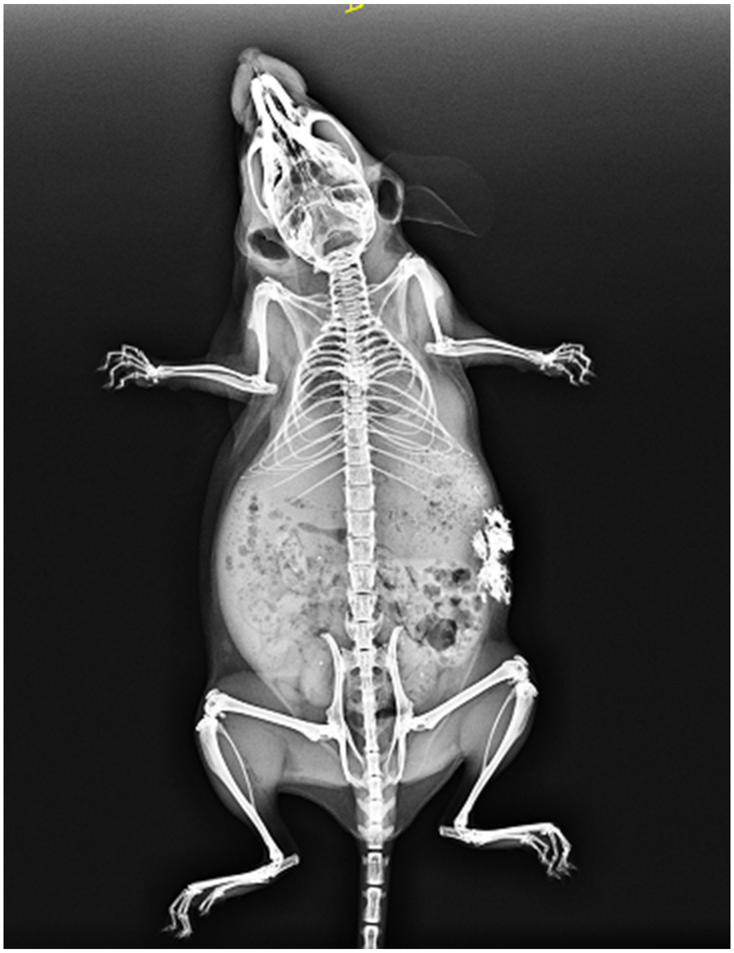

Supplementary Figure S1: X-ray imaging of mouse implanted with human bone. The image show the presence of a small fragment of human bone implanted SC in the flank, and it does not reveal lesions in the bones of the mouse.

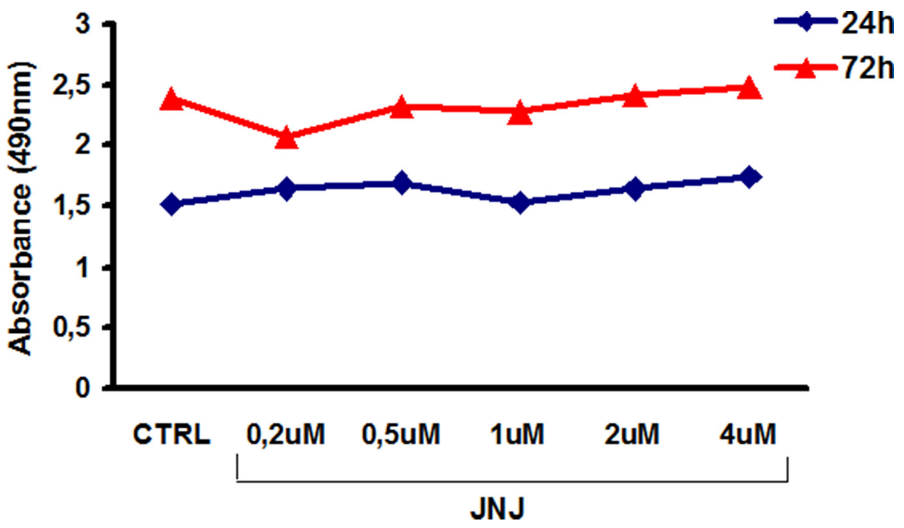

Supplementary Figure S2: MTT assay. At different time points (24 and 72 hours) JNJ was not toxic on RCC stem cell culture *in vitro*.

Supplementary Table S1: Patients' characteristics

Histology	Sex	Age	TNM
ccRCC	M	51	pT1a-Nx
ccRCC	F	51	pT3a-N0
pRCC	M	33	pT1a-N0
ccRCC	M	70	pT2- Nx
ccRCC	M	59	pT1a-Nx
ccRCC	F	48	pT3a-Nx
ccRCC	M	73	pT1a-Nx
ccRCC	F	67	pT1a-Nx
ccRCC/pRCC	M	67	pT3a-Nx
crRCC	M	69	pT1a-Nx
ccRCC	F	82	pT3a-Nx
ccRCC	M	65	pT1a-Nx
ccRCC	M	54	M1
ccRCC	M	48	M1
ccRCC	M	74	M1
ccRCC	F	52	M1

ccRCC: clear cell Renal Cell Carcinoma; pRCC: papillary Renal Cell carcinoma.
